# 412. Estimating the Impact of School Classroom Sizes on the Probability of Severe Acute Respiratory Syndrome Coronavirus-2 Infectivity or Exposure

**DOI:** 10.1093/ofid/ofab466.613

**Published:** 2021-12-04

**Authors:** Sanya J Thomas, Rebecca R Young, Ibukunoluwa Akinboyo, Michael J Smith, Tara Buckley, Sarah S Lewis

**Affiliations:** 1 Nationwide Children’s Hospital/OSU, Columbus, Ohio; 2 Duke University Medical Center, Durham, North Carolina; 3 Duke University, Durham, North Carolina; 4 Durham County Public Health Department, Durham, North Carolina

## Abstract

**Background:**

Despite schools reopening across the United States in communities with low and high Coronavirus disease 2019 (COVID-19) prevalence, data remain scarce about the effect of classroom size on the transmission of severe acute respiratory syndrome coronavirus-2 (SARS-COV-2) within schools. This study estimates the effect of classroom size on the risk of COVID-19 infection in a closed classroom cohort for varying age groups locally in Durham, North Carolina. Total number of Coronavirus Disease 2019 (COVID-19) infections over a 28-day follow-up period for varying classroom reproduction number (R0) and varying classroom cohort sizes of 15 students, 30 students and 100 students in Durham County, North Carolina.

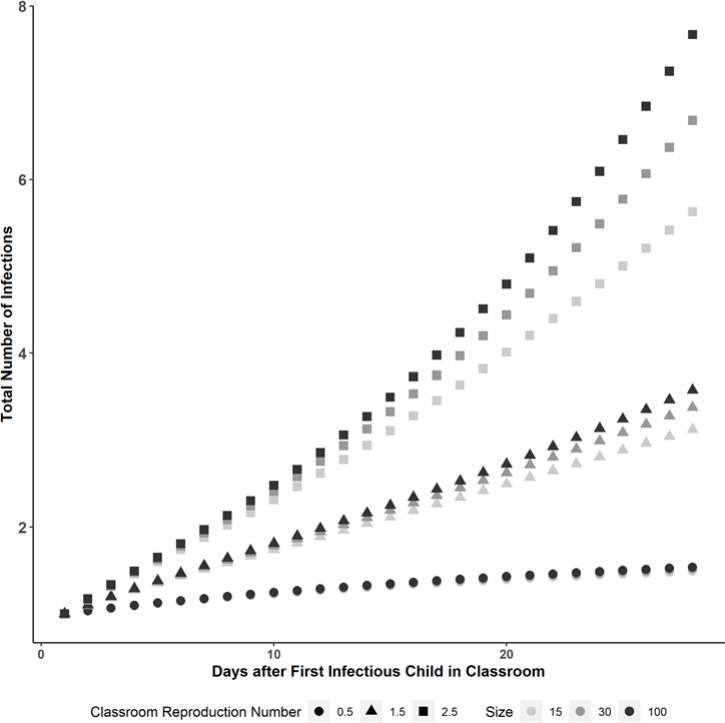

**Methods:**

Using publicly available population and COVID-19 case count data from Durham County, we calculated a weekly average number of new confirmed COVID-19 cases per week between May 3, 2020 and August 22, 2020 according to age categories: < 5 years, 5-9 years, 10-14 years, and 15-19 years. We collated average classroom cohort sizes and enrollment data for each age group by grade level of education for the first month of the 2019-2020 academic school year. Then, using a SEIR compartmental model, we calculated the number of susceptible (S), exposed (E), infectious (I) and recovered (R) students in a cohort size of 15, 30 and 100 students, modelling for classroom reproduction number (R_0_) of 0.5, 1.5 and 2.5 within a closed classroom cohort over a 14-day and 28-day follow-up period using age group-specific COVID-19 prevalence rates.

**Results:**

The SEIR model estimated that the increase in cohort size resulted in up to 5 new COVID-19 infections per 10,000 students whereas the classroom R_0_ had a stronger effect, with up to 88 new infections per 10,000 students in a closed classroom cohort over time. When comparing different follow-up periods in a closed cohort with R_0_ of 0.5, we estimated 12 more infected students per 10,000 students over 28 days as compared to 14 days irrespective of cohort size. With a R_0_ of 2.5, there were 49 more infected students per 10,000 students over 28 days as compared to 14 days.

**Conclusion:**

Classroom R_0_ had a stronger impact in reducing school-based COVID-19 transmission events as compared to cohort size. Additionally, earlier isolation of newly infected students in a closed cohort resulted in fewer new COVID-19 infections within that group. Mitigation strategies should target promoting safe practices within the school setting including early quarantine of newly identified contacts and minimizing COVID-19 community prevalence.

**Disclosures:**

**Michael J. Smith, MD, M.S.C.E**, **Merck** (Grant/Research Support)**Pfizer** (Grant/Research Support)

